# Publisher Correction: Feature-based attention enables robust, long-lasting location transfer in human perceptual learning

**DOI:** 10.1038/s41598-021-96732-7

**Published:** 2021-08-23

**Authors:** Shao-Chin Hung, Marisa Carrasco

**Affiliations:** 1 Department of Psychology, New York University, New York, NY USA; 2 Center for Neural Science, New York University, New York, NY USA

Correction to: *Scientific Reports* 10.1038/s41598-021-93016-y, published online 06 July 2021

The original version of this Article contained an error in the Discussion section, where Reference 81 was incorrectly cited as Reference 88.

“The Reverse Hierarchy Theory^41,88^ predicts specificity in difficult tasks in which training modifies low-level, location- and feature-specific units, and transfer in easy tasks in which training-induced modifications are at high-level, broadly-tuned units.”

now reads:

“The Reverse Hierarchy Theory^41,81^ predicts specificity in difficult tasks in which training modifies low-level, location- and feature-specific units, and transfer in easy tasks in which training-induced modifications are at high-level, broadly-tuned units.”

The original Article has been corrected.

